# Synthesis and Characterization of Molybdenum- and Sulfur-Doped FeSe

**DOI:** 10.1021/acsomega.3c05684

**Published:** 2023-09-18

**Authors:** Marwa H.A. Aouelela, Mohamed Taha, Samaa I. El-dek, Abdelwahab Hassan, Alexander N. Vasiliev, Mahmoud Abdel-Hafiez

**Affiliations:** † Materials Science and Nanotechnology Department, Faculty of Postgraduate Studies for Advanced Sciences, Beni-Suef University, 62511 Beni-Suef, Egypt; ‡ Department of Physics, Faculty of Science, Fayoum University, 63514 Fayoum, Egypt; § National University of Science and Technology MISiS, 119049 Moscow, Russia; ∥ Lomonosov Moscow State University, 119991 Moscow, Russia; ⊥ Department of Physics and Astronomy, Uppsala University, Box 516, SE-75120 Uppsala, Sweden; # Department of Applied Physics and Astronomy, University of Sharjah, P. O. Box 27272 Sharjah, United Arab Emirates

## Abstract

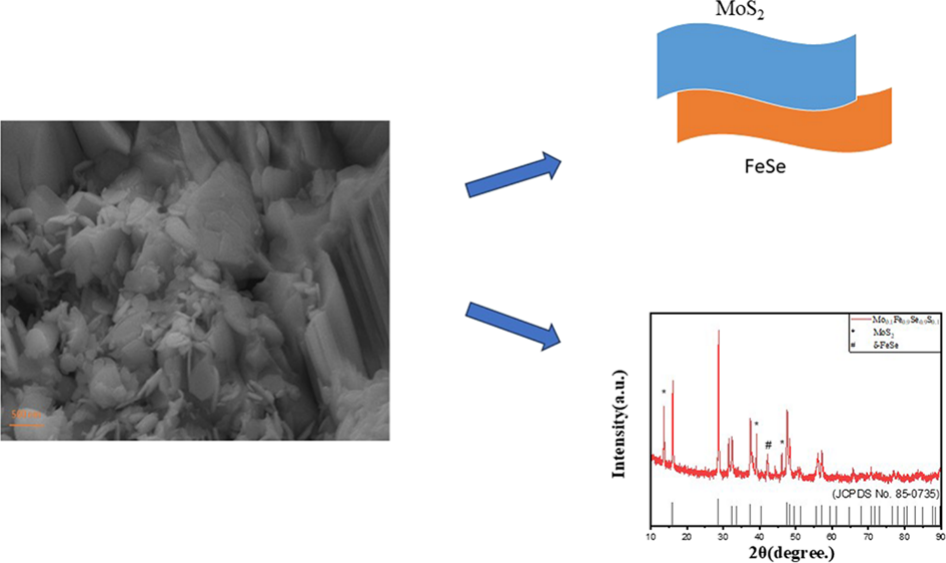

During the past decade, two-dimensional (2D) layered materials opened novel opportunities for the exploration of exciting new physics and devices owing to their physical and electronic properties. Among 2D materials, iron selenide has attracted much attention from several physicists as they provide a fruitful stage for developing new superconductors. Chemical doping offers a powerful approach to manipulate and optimize the electronic structure and physical properties of materials. Here, to reveal how doping affects the physical properties in FeSe, we report on complementary measurements of molybdenum- and sulfur-doped FeSe with theoretical calculations. Mo_0.1_Fe_0.9_Se_0.9_S_0.1_ was synthesized by a one-step solid-state reaction method. Crystal structure and morphology were studied using powder X-ray diffraction and scanning electron microscopy. Thermal stability and decomposition behavior in doped samples were studied by thermogravimetric analysis, and to understand the microscopic influence of doping, we performed Raman spectroscopy. First-principles calculations of the electronic structure illustrate distinct changes of electronic structures of the substituted FeSe systems, which can be responsible for their superconducting properties.

## 1 Introduction

Chemical doping has proven to be an effective tool for tailoring and manipulating the electronic properties of various materials. Since the discovery of iron-based superconductors (IBSCs), the excellent and distinctive properties of IBSCs have attracted a lot of attention, such as the resilience to impurity doping, high upper critical field (B_c2_), and high critical temperature (*T*_c_).^1−3^ One of the ideal systems in IBSCs to probe the mechanism of superconductivity and to search for high-temperature superconductors is the iron selenide (FeSe). Chemical doping in FeSe involves substituting some of the Fe or Se atoms with different elements or compounds. This process modifies the electronic structure and introduces new electronic interactions, leading to changes in the material’s physical properties. Additionally, FeSe, the simplest composition of the IBSC family, is an ideal platform to study the nematic phase with a wide temperature range, and FeSe is considered to be the least toxic starting material.^4^ Superconductivity without doping appears in FeSe with a temperature (*T*_c_) of ∼8 K within β-FeSe and is enhanced under high pressure up to 37K.^5^ FeSe has a structural transition at *T*_s_ ∼87 K from the tetragonal-to-orthorhombic phase like other iron-based families by decreasing temperature, but unlike the other iron-based systems, this structure transition is not followed by magnetic order.^6−9^ Overall, the nature and the electronic ordering boundary and superconductivity in most of the quasi-two-dimensional materials are still attractive problems. Particularly, in FeSe, the relation between superconductivity and nematicity is an unsolved question compared to the IBSCs-122-family.^10−12^ One way to study the physical properties in quasi-two-dimensional materials is to investigate the effect of chemical doping. Therefore, many researchers try to use different elements of doping to replace Fe or Se sites.^11^ Among the variety of possible substitutions, transition-metal (TM) ion substitution at the Fe site is the most informative with regard to questions such as the pairing symmetry and the nature of the low-energy excitation TM doped FeSe like Al, Ni, Co, Te, Ga, Cr, In, Ba, and Sm.^12^ Some of the transition metals are doped into the β-FeSe structure, generally below 12 at. %. However, the prepared materials are single phase, but no intrinsic increase in the *T*_c_ was observed. On the other hand, at the high level of substitution, i.e., over 25 at. %, superconductivity was suppressed.^12,13^ For S-doped FeSe, under high pressure, the critical nematic fluctuations vanish, whereas magnetic order is dramatically induced.^14,15^ Unlike FeSe under physical pressure, isoelectronic S substitution Se site can tune *T*_s_ to a nematic critical point (NCP) at *x* ≈ 0.17 with abrupt change of superconductivity, but no magnetic order appears in FeSe_1–*x*_S_*x*_ single crystals under ambient pressure. Recently, by simultaneously tuning chemical and physical pressures, a strikingly enhanced *T*_c_ has been obtained near both ends of the dome-shaped spin density wave (SDW) phase in FeSe_1–*x*_S_*x*_ rather than near the NCP.^16−22^ There are many methods for the synthesis of FeSe materials, and these can be divided into four groups: the one step method, the flux method, the high-pressure method, and the solid-state reaction method.^17^ The third and last methods are usually used to prepare polycrystalline samples, and the others are used for single crystal preparation.^18,19^ Generally, it is very difficult to prepare single crystal samples of FeSe with different ratios because of its sensitivity to stoichiometry.^20^

In this work, we have synthesized a series of iron selenides, namely, FeSe_0.9_, Fe_0.9_Se_0.9_, FeSe_0.9_S_0.1_, and Mo_0.1_Fe_0.9_Se_0.9_S_0.1_, by a one-step solid-state reaction. From the reported literature, the S-doped FeSe with 10% in the Se site can enhance the superconductivity of FeSe, and the transition-metal-doped FeSe in the Fe site with a very low molar ratio slightly raises the critical temperature. Several characterization techniques were employed to analyze the samples including X-ray diffraction (XRD), scanning electron microscopy (SEM) connected with energy-dispersive X-ray spectroscopy (EDS), and Raman spectroscopy. In addition to the experimental work, we made some DFT calculations to better understand the doping effect of Mo on both the density of state and band structure of FeSe.

## 2 Materials and Methods

For S-doped FeSe, we prepared it using two methods. The first method was by a one-step solid-state reaction similarly to FeSe and Mo-doped samples. In the second method, after the growth of the β-FeSe phase by a one-step method, we reground the FeSe pellets in the glove box by adding S by 10% of weight and then mixed it together for 30 min. The obtained mixture was cold pressed again into pellets (6 mm diameter, 1.5 mm thick, 0.4 g) using a uniaxial pressure of 10 MPa. The pellet was completely sealed inside an evacuated quartz ampoule and then heated at 650 °C for 24 h.^21^

For polycrystalline FeSe doped samples with Mo and S, samples were prepared by a one-step solid-state reaction, with starting materials Fe (99.9% purity), Se (99.5% purity), S (99.5% purity), and Mo (99.9% purity, from Sigma) powders. With different molar ratios, the powders were mixed together in the agate mortars inside the glove box for more than 30 min. The mixture was then compressed into pellets (6 mm diameter, 1.5 mm thick, 0.4 g) using a uniaxial pressure of 10 MPa.^22^ The pellets were sealed inside an evacuated quartz ampoule. The ampoule was then heated to 700 °C with a heating rate of 50 °C/h and maintained there for 1 h. Then, to grow the tetragonal phase of FeSe, the temperature was reduced to 400 °C in 30 min and held for 40 h. Finally, to avoid phase transformation at low temperature, the quartz ampoule with the samples was quenched in the air to reach room temperature within 5 min.^20,23^

In this paper, there are two major phases of the FeSe structure. The first phase is the tetragonal β-FeSe phase, characterized by layers of FeSe_4_ tetrahedra that share edges with the *P*4/*nmm* space group. The second phase is the hexagonal δ-FeSe phase. It is well known that only the tetragonal phase exhibits superconducting properties. For our computational calculations, we utilized the density functional theory (DFT) using the CASTEP code in Materials Studio.^24^ The exchange correlation function was treated using the local density approximation (LDA) parameterized by Ceperley–Alder.^25^ A 600 eV energy cutoff was chosen. Ultrasoft pseudopotentials were applied, the electronic energy tolerance of the self-consistent field (SCF) was selected as 1.0 × 10^–6^ eV, and the K point of 10 × 10 × 8 was set as non-spin polarized.^26^

### 2.1 Characterization

A sequence of characterizations has been performed for samples to confirm the structure and observe the properties of the samples. A scanning electron microscope (ZEISS Sigma 500 VP, coupled with EDS) was used to investigate morphology of polycrystalline samples. X-ray diffraction (PANalytical Empyrean, Netherlands) was used to determine the crystal. Thermogravimetric analysis (TGA, STA6000, PerkinElmer) was used to study the weight changes of a sample as a function of temperature Raman spectroscopy, which provides the information of molecules, phase, and the chemical bond (WITec alpha 300 RA confocal Raman microscopes) with 50× objective. Excitation was performed for each sample by a 53 2 nm Nd:YAG laser.

## 3 Results and Discussion

### 3.1 X-ray Diffraction

**Figure 1:**
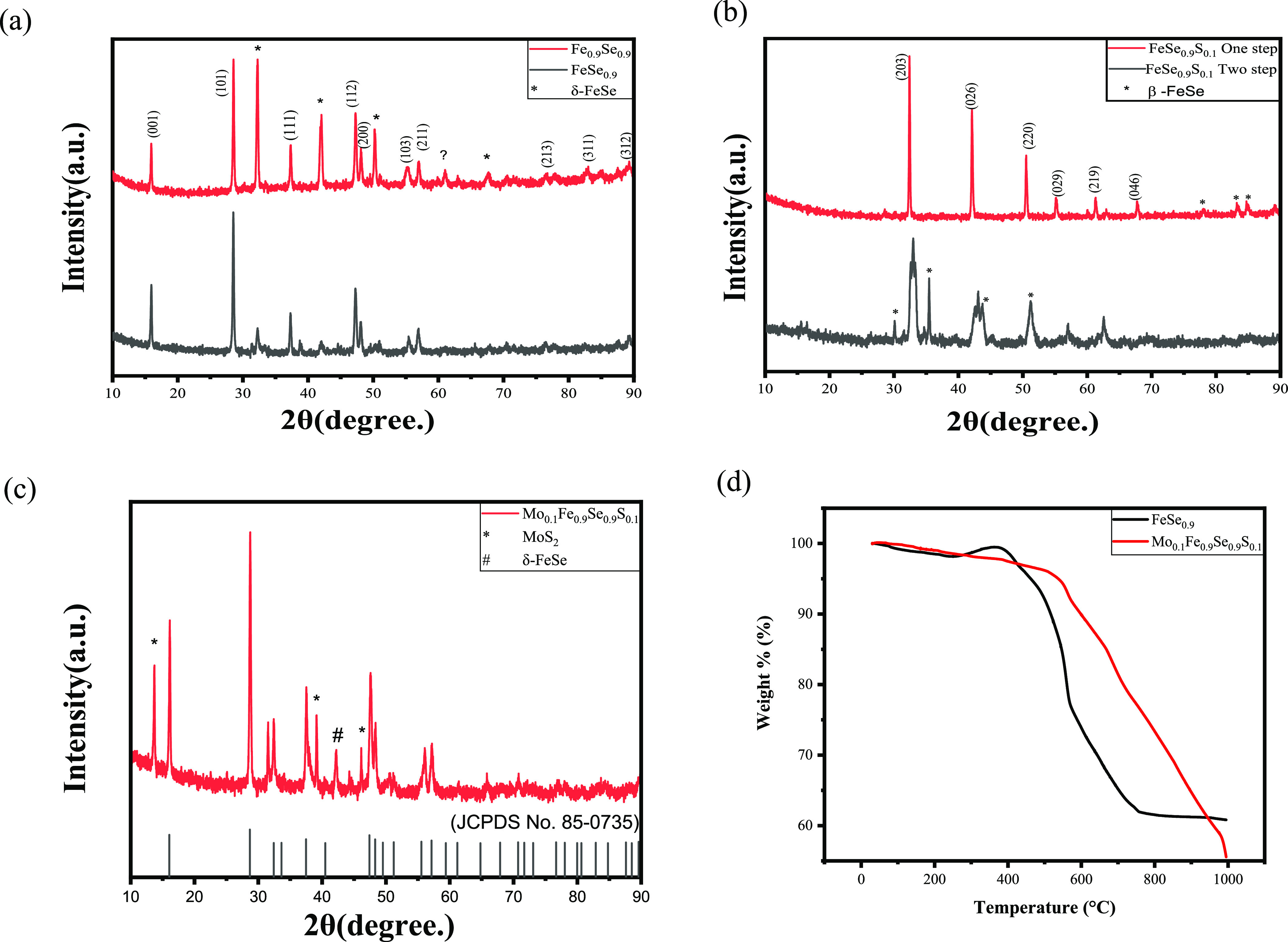
X-ray diffraction pattern of (a) FeSe_0.9_ and Fe_0.9_Se_0.9_, (b) FeSe_0.9_S_0.1_ sample with one- and two-step methods, and (c) Mo_0.1_Fe_0.9_Se_0.9_S_0.1_ with 2θ range of 10–90 and (d) TGA curves of the Mo_0.1_Fe_0.9_Se_0.9_S_0.1_ and FeSe_0.9_ samples.

In Figure 1a, the X-ray powder diffraction patterns at room temperature of Fe/Se (1:0.9 and 0.9:0.9) are shown. For FeSe_0.9_ in which β-FeSe (JCPDS No. 85-0735) is the primary phase (∼90% by volume), the XRD pattern is well indexed using the JCPDS card for the predominant space group of *P*4/*nmm*. High-intensity peaks of tetragonal FeSe are related to (011), (101), and (122) planes at 2θ = 15.8468, 28.4895, and 47.2949, respectively, which belong to the superconducting phase in the FeSe system. By melting at high temperature, the hexagonal phase of FeSe is formed after cooling down below 457 °C; the phase transition from the hexagonal structure to the wanted tetragonal phase of FeSe is always expected at 400 °C. A very small amount of Fe_7_Se_8_ was observed and indexed according to the JCPDS file (No. 71-0586) to be the hexagonal phase. For the Fe_0.9_Se_0.9_ sample, asterisks indicated the hexagonal phase of FeSe. By comparing the XRD patterns of both samples, one can notice how the molar ratio between Fe and Se greatly affects the tetragonal/hexagonal phase. With decreasing Fe, the probability of formation of the hexagonal phase of FeSe increases.

In Figure 1b, the X-ray powder diffraction pattern of FeSe_0.9_S_0.1_ is shown. The δ-FeSe (Fe_7_Se_8_; JCPDS No. 71-0586) is the primary phase (around 80% by volume). In the case of FeSe_0.9_S_0.1_ prepared by the one-step method, the structure is totally hexagonal with no presence of a β-phase. On the other hand, in the FeSe_0.9_S_0.1_ prepared by the two-step method, a mixed hexagonal and tetragonal phase exists. The peak splitting of FeSe doped sulfur by two steps is clear, with the appearance of the tetragonal phase when comparing the pattern by JCPDS No. 85-0735. In Figure 1c, the X-ray powder diffraction pattern of Mo_0.1_Fe_0.9_Se_0.9_S_0.1_ is shown, in which β-FeSe (JPCDS No. 85-0735) is the primary phase (around 70% by volume). Asterisks indicate the MoS_2_ phase and well indexed with the standard JCPDS card no. 37-1492 (space group *P*63/*mmc*, No. 194). The reflections located at 2θ = 13.7321 and 39.1075 were indexed to be the (002) and (103) planes, respectively, corresponding to the hexagonal phase of MoS_2_.^27^ Other peaks were determined as the tetragonal phase. We can see that a very small amount of Mo atoms enters the FeSe system and that a larger percentage of Mo atoms does not enter the FeSe system; instead, MoS_2_ is grown layer-by-layer on FeSe. One of the main reasons behind the solubility limit of Mo inside the FeSe matrix could be the high atomic radius of Mo atoms compared to Fe atoms. The shifts in peak positions, specifically the (011) and (101) peaks, can indeed be attributed to the successful incorporation of sulfur and molybdenum into the FeSe crystal lattice. This phenomenon results in alterations in the unit cell parameters and atomic arrangements, subsequently leading to shifts in the diffraction pattern.^28^

### 3.2 Thermogravimetric Analysis

In the case of FeSe, TGA can be used to investigate its thermal stability and decomposition behavior. FeSe consists of iron and selenium; as shown in Figure 1d, it is stable up to 500 °C for the tetragonal phase of FeSe but starts to decompose above that temperature for the NiAs structure, and this confirms our preparation conditions for the tetragonal phase of FeSe. Therefore, TGA of FeSe can be performed in the temperature range of 25–1000 °C under a nitrogen atmosphere. The weight loss can be attributed to the decomposition of FeSe, where the selenium is released as a gas^29^ and the iron remains as a solid residue. The amount of weight loss of around 40% of the total mass can be used to determine the stoichiometry of the decomposition reaction. The TGA curve of FeSe typically shows a weight loss of around 10–15% in the temperature range of 500–600 °C, which corresponds to the decomposition of FeSe into Fe and Se. The observed weight gain in the FeSe_0.9_ sample within the temperature range of 300–400 °C is attributed to the formation of Fe_2_O_3_.^30^ In contrast, the Mo_0.1_Fe_0.9_Se_0.9_S_0.1_ sample demonstrates stability up to 400 °C, primarily attributed to the presence of FeSe/MoS_2_. However, beyond this temperature, the Mo_0.1_Fe_0.9_Se_0.9_S_0.1_ sample undergoes decomposition, primarily due to sulfur evaporation. It is noteworthy to mention that prior research, specifically the work of Pandy et al.,^33^ has documented the stability of MoS_2_ at elevated temperatures, specifically above 1000 °C. In our current study, we have deliberately chosen to focus on presenting the thermal stability of our findings within a temperature range spanning from 25 to 1000 °C. This approach allows us to comprehensively investigate the behavior of the materials under conditions relevant to practical applications and experimental constraints.^32,33^

### 3.3 Scanning Electron Microscopy

**Figure 2:**
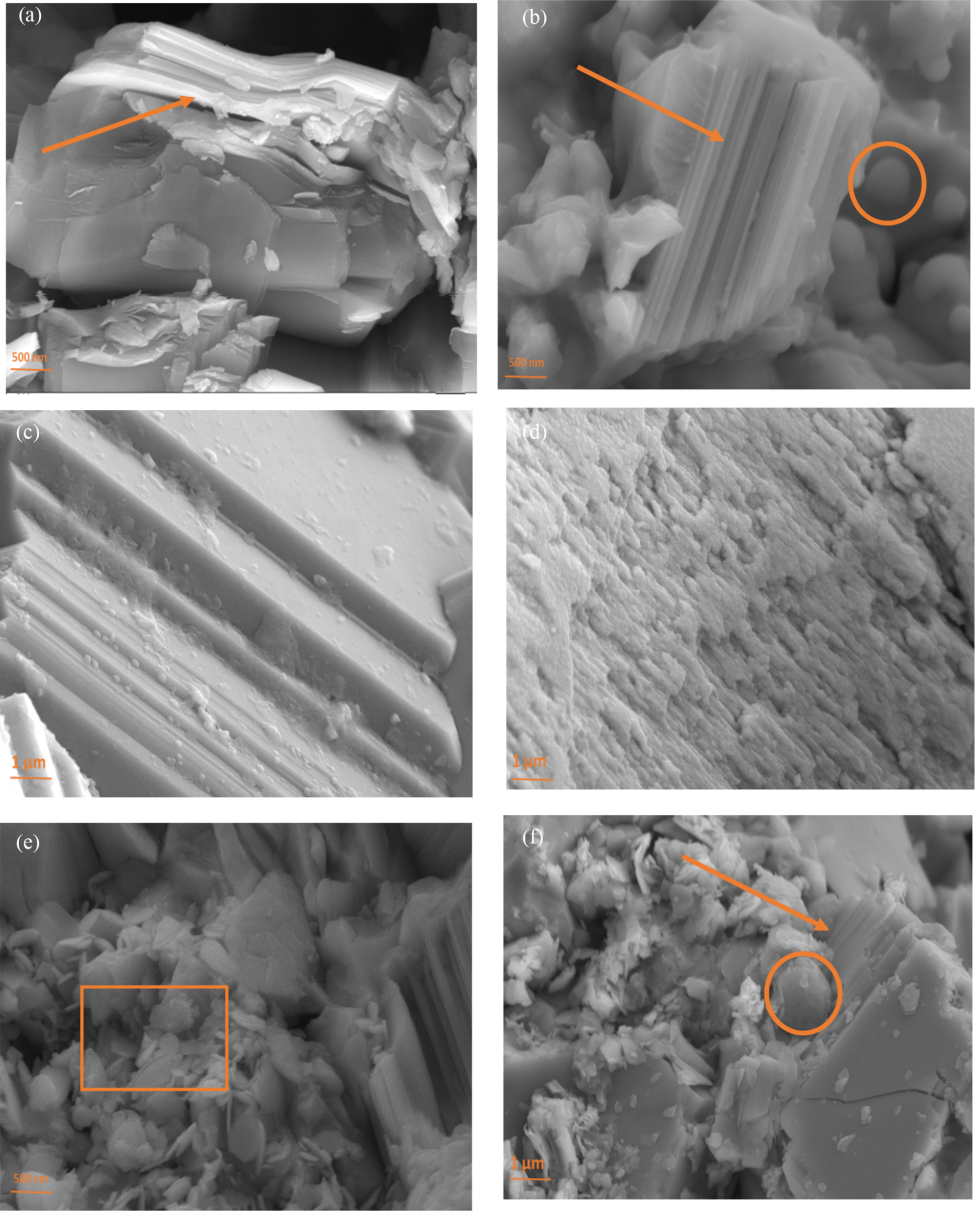
FESEM images (a) FeSe_0.9_, (b) Fe_0.9_Se_0.9_, (c) FeSe_0.9_S_0.1_ prepared in two steps, (d) FeSe_0.9_S_0.1_ prepared in one step, and (e, f) Mo_0.1_Fe_0.9_Se_0.9_S_0.1_ sample_._

The SEM images in Figure 2 (FESEM) of (a) FeSe_0.9_ and (b) Fe_0.9_Se_0.9_ samples show the layered structure of the main tetragonal phase with increasing probability of the hexagonal phase of FeSe due to the Fe vacancy, which agrees with the XRD pattern. Figure 2c shows the FESEM image of FeSe_0.9_S_0.1_ prepared by the two-step solid-state reaction method. Figure 2d shows FeSe_0.9_S_0.1_ prepared by the two-step solid-state reaction method; the point remark is the existence of layers for the two preparation techniques as well as the preferred orientation of layers. The differences between the two methods are the layer domain and layer spacing. The hexagonal morphology seems to be present in the two samples. The one prepared with two steps has a more perfect crystalline morphology with less defects and better ordering. Figure 2e,f shows the FESEM image of Mo_0.1_Fe_0.9_Se_0.9_S_0.1_ showing enlarged grains, which seem to connect more tightly with each other than the undoped one. The Mo can accelerate the transformation from Fe_7_Se_8_ to tetragonal FeSe and the growth of grains by improving atomic diffusion, thus making the sintering process much faster than that in the undoped sample. The layered structure of the two samples that belong to the tetragonal phase is clear and confirms the XRD results with the presence of spherical shapes and flakes related to Fe_7_Se_8_ and MoS_2_.^28^

**Figure 3:**
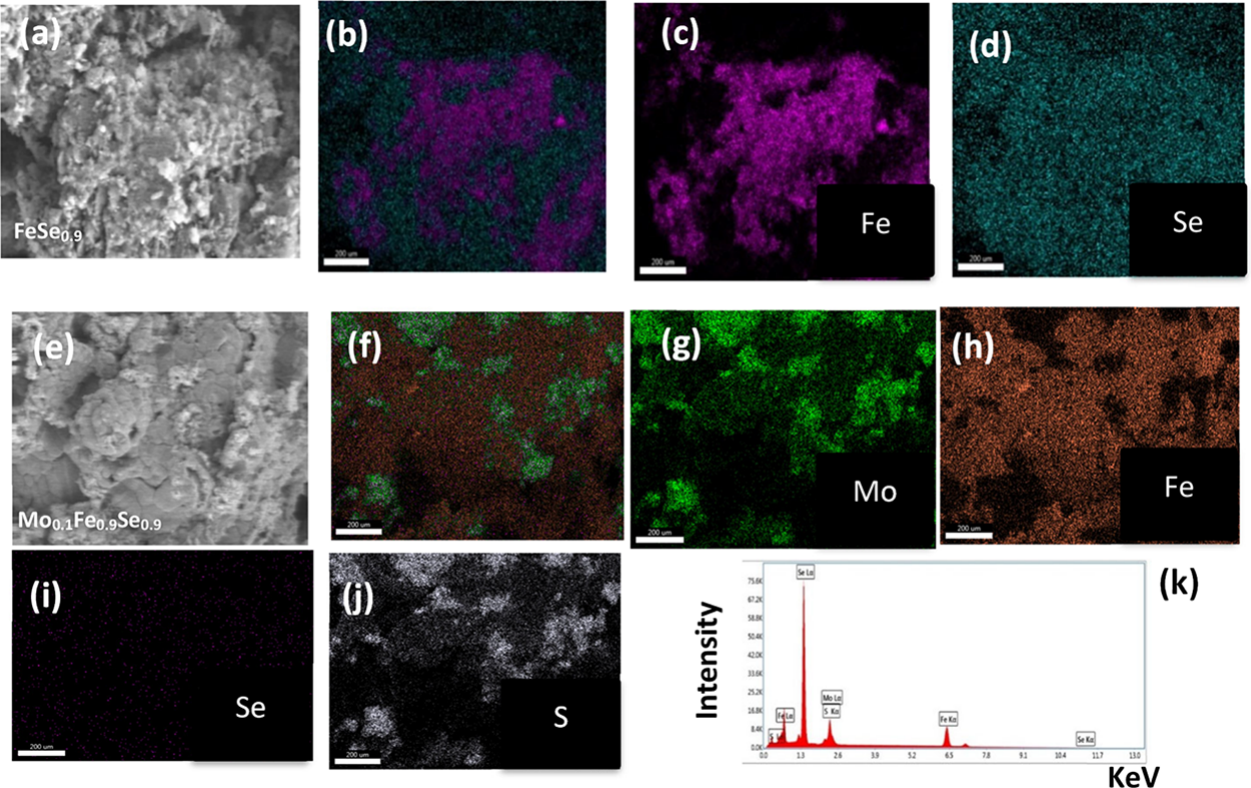
FESEM of (a, b) FeSe_0.9_. Compositional mappings of (c) Fe, (d) Se, and (e) Mo_0.1_Fe_0.9_Se_0.9_S_0.1_. Compositional mappings of (f) Mo_0.1_Fe_0.9_Se_0.9_S_0.1_, (g) Mo, (h) Fe, (i) Se, and (j) S. (k) EDX spectra.

In Figure 3, we provide an insightful compositional mapping, which enables us to evaluate the distribution of different elements within both undoped FeSe_0.9_ and Mo_0.1_Fe_0.9_Se_0.9_S_0.1_ samples. This mapping provides valuable information regarding the spatial distributions of Fe, Se, Mo, and sulfur within the samples, thus confirming the molar ratio of the starting materials employed in the experiment. For the Mo_0.1_Fe_0.9_Se_0.9_S_0.1_ sample, we can observe that Mo and S are not uniformly distributed because of the presence of two phases of MoS_2_ and FeSe confirmed by PXRD.

### 3.4 Raman Spectroscopy

**Figure 4:**
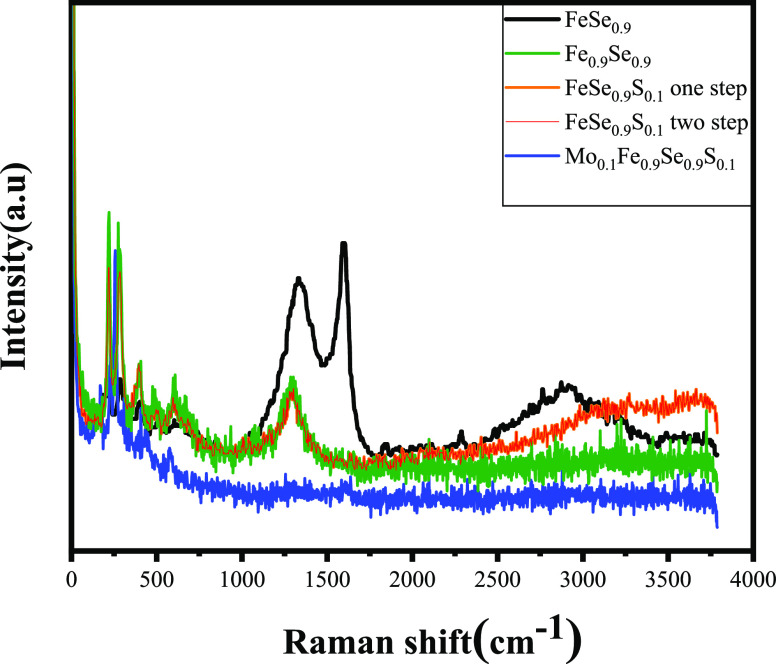
Raman spectra for FeSe_0.9_, Mo-doped FeSe_0.9_, and FeSe_0.9_S_0.1_.

The Raman spectroscopic study for FeSe doped S and Mo materials is shown in Figure 4 to understand the microscopic influence of the doping. However, the origin of this mode observed in the Raman spectrum remains unclear and needs further investigation. The surface states in FeSe prepared by the solid-state reaction at room temperature are still unsolved. FeSe materials with Fe and Se atoms are occupying associated Wyckoff positions 2c and 2a. The previous study reported by Kumar et al.^34^ showed that the Raman spectra of polycrystalline FeSe_0.82_ exhibit seven active modes. Four of them are distinguished as Eg: 106 cm for Se, 160 cm for A1g, 224 cm for Eg, and 234 cm for B1g for Fe. The Raman peak at 254 cm^–1^ belongs to the δ-FeSe phase. In our results, there are four Raman active phonon modes appearing related to A_1g_ + B_1g_ + 2E_g_.^14^Figure 4 shows the Raman spectrum at room temperature (wavelength = 512 nm), which is divided into two parts based on the spectral range. The low-frequency range (80–350 cm^–1^) shows more than five Raman bands assigned to the main tetragonal structure containing more than 10% hexagonal phases of Fe_7_Se8_8_. The strong Raman bands are in the high-frequency range (800–1800 cm^–1^). In the high-frequency modes from 1300 to 1600 cm^–1^, two peaks could be assigned to the electronic Raman scattering of the d-orbitals of Fe as reported by Okazaki et al.^35^ Our XRD and Raman results agree with this consideration. For FeSe_0.9_, two strong Raman bands appear that belong to the d-orbitals. The S-doped samples show one peak related to Fe d-orbitals around 1300 cm^–1^. For Mo doping, we have just week peaks indicating the vacancy of Fe and d-orbital disorder. The peak appearing at 406 cm^–1^ points to the existence of MoS_2_ peaks as the separated secondary phase. This was a direct consequence of the low solubility limit (10% of the total mass) with the FeSe lattice as mentioned depending on preparation conditions.

This secondary phase is identified from XRD patterns and well visualized from FESEM on the grains boundaries as flakes on the surface FeSe layers. As shown by Kumar et al., FeSe is temperature dependent, and the FeSe Raman active modes are at 1 K. The Raman band around 3000 cm^–1^ is assigned to the 2D materials. The new peak at 257 cm^–1^ for the Mo_0.1_Fe_0.9_Se_0.9_S_0.1_ sample can be attributed to the presence of the oxide admixture in the Fe/Se/MoS_2_ polycrystalline system. Therefore, the relatively higher intensity peaks of impurity phases in the Raman spectrum do not necessarily indicate a higher impurity content in the prepared FeSe. The higher intensity bands of impurity in the samples could be due to the fact that the covalently bonded oxide states are more Raman sensitive as compared to the relatively weak Raman active Fe-Se related vibrations. In other words, the Raman scattering cross section of oxide-related vibrations is larger than that of Fe-Se related vibrations.^33^

### 3.5 Optimized Structure and Calculated Band Structure

**Figure 5:**
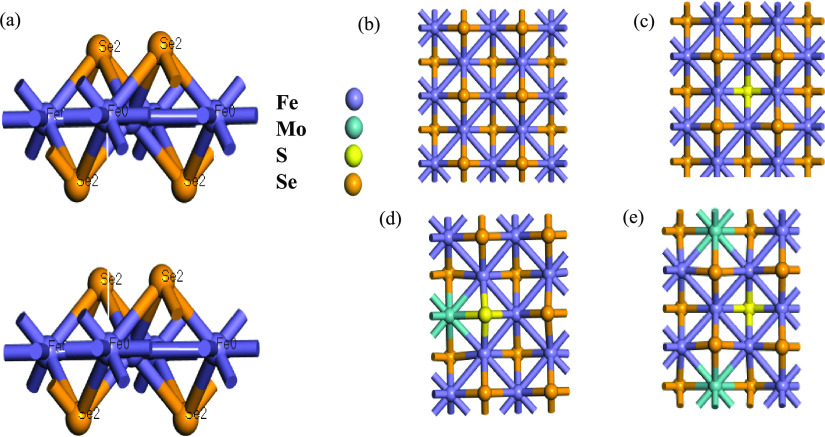
(a) Optimized structures of tetragonal FeSe (space group *P*4/*nmm*). The *xy*-projection of 2 × 2 × 1 supercells used for (b) FeSe, (c) FeSe_0.875_S_0.125_, (d) Mo_0.125_Fe_0.875_Se_0.875_S_0.125_ with symmetry position (1), and (e) Mo_0.125_Fe_0.875_Se_0.875_S_0.125_ with symmetry position (2).

First, we performed a geometry optimization to the FeSe unit cell as shown in Figure 5. In panel a, the lattice parameters of the tetragonal phase of FeSe (129 *P*4/*nmm*) were very close to the experimental lattice parameters *a* = 3.765 Å and *c* = 5.518 Å. Second, in the case of partially substituted systems, we used the 2 × 2 × 1 super cell in fig(b) , In Figure 5(d,e) 12.5 at. % of Mo in Fe site and 12.5 at. % of S in Se site as appears in Figure 5 (c). With non-spin polarized.^34^ This was equivalent to the prepared chemical formula.

**Figure 6:**
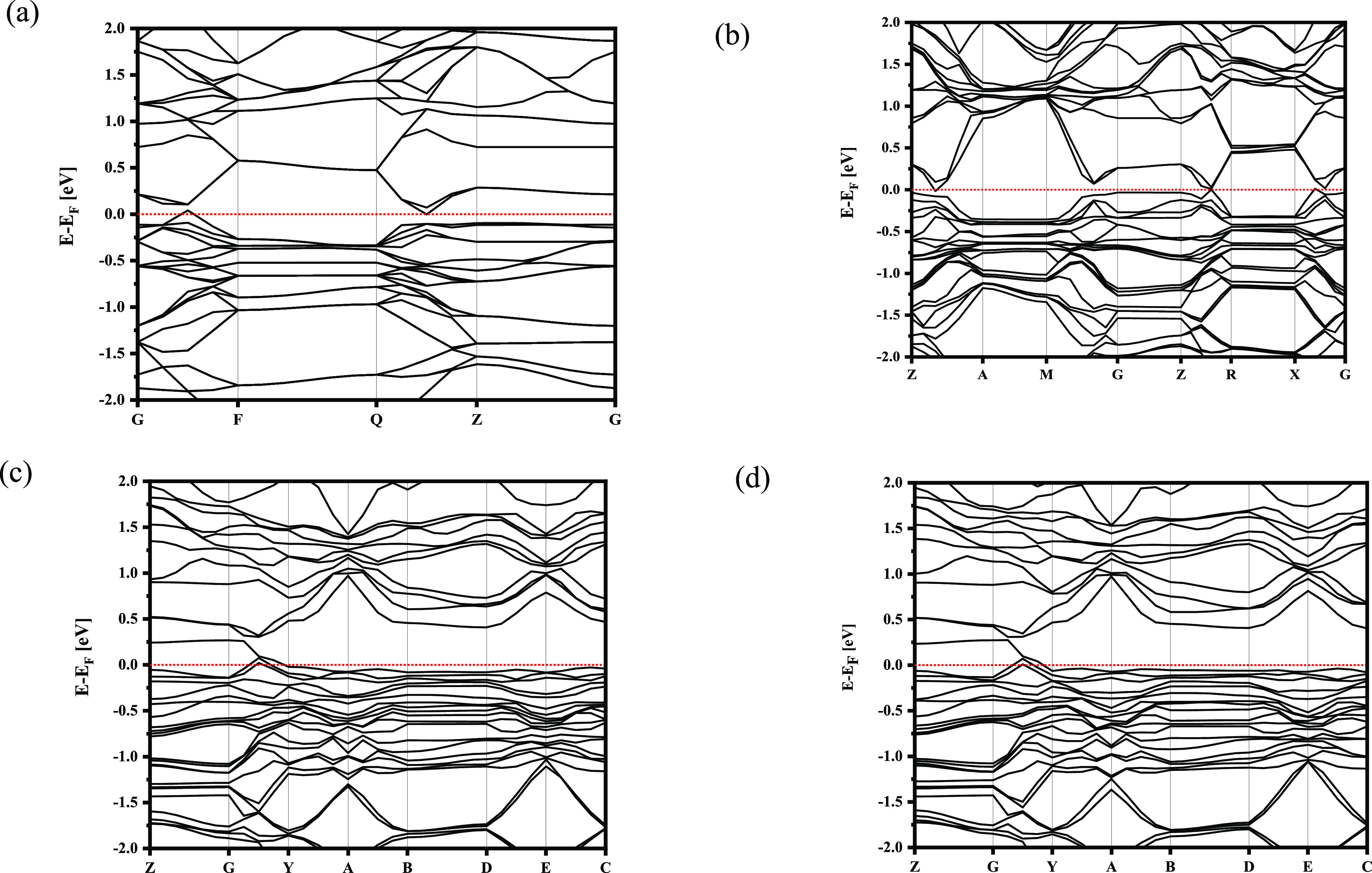
Band structure of (a) FeSe, (b) FeSe with 12.5% doping concentration of sulfur in Se ion site, and (c, d) Mo_0.125_Fe_0.875_Se_0.875_S_0.125_ in different symmetry position (1, 2), respectively.

The calculated non-spin polarized LDA band structure of FeSe, Mo, and S doped FeSe is presented in Figure 6. The figure prominently demonstrates that substituting sulfur atom for the Se site and Mo for the Fe site results in significant changes to the band structure near the Fermi level. Interestingly, the band structure remains unchanged in the symmetry position of Mo within the crystal structure, as observed from the consistent band structures shown in panels c and d.^31^ Additionally, the band structure of Mo_0.125_Fe_0.875_Se_0.875_S_0.125_ appears flatter when compared to S-doped FeSe in Figure 6b around the Fermi surface. In the case of Mo substitution, this distinctive flat band structure has the potential to increase the density of states at specific energy levels.

**Figure 7:**
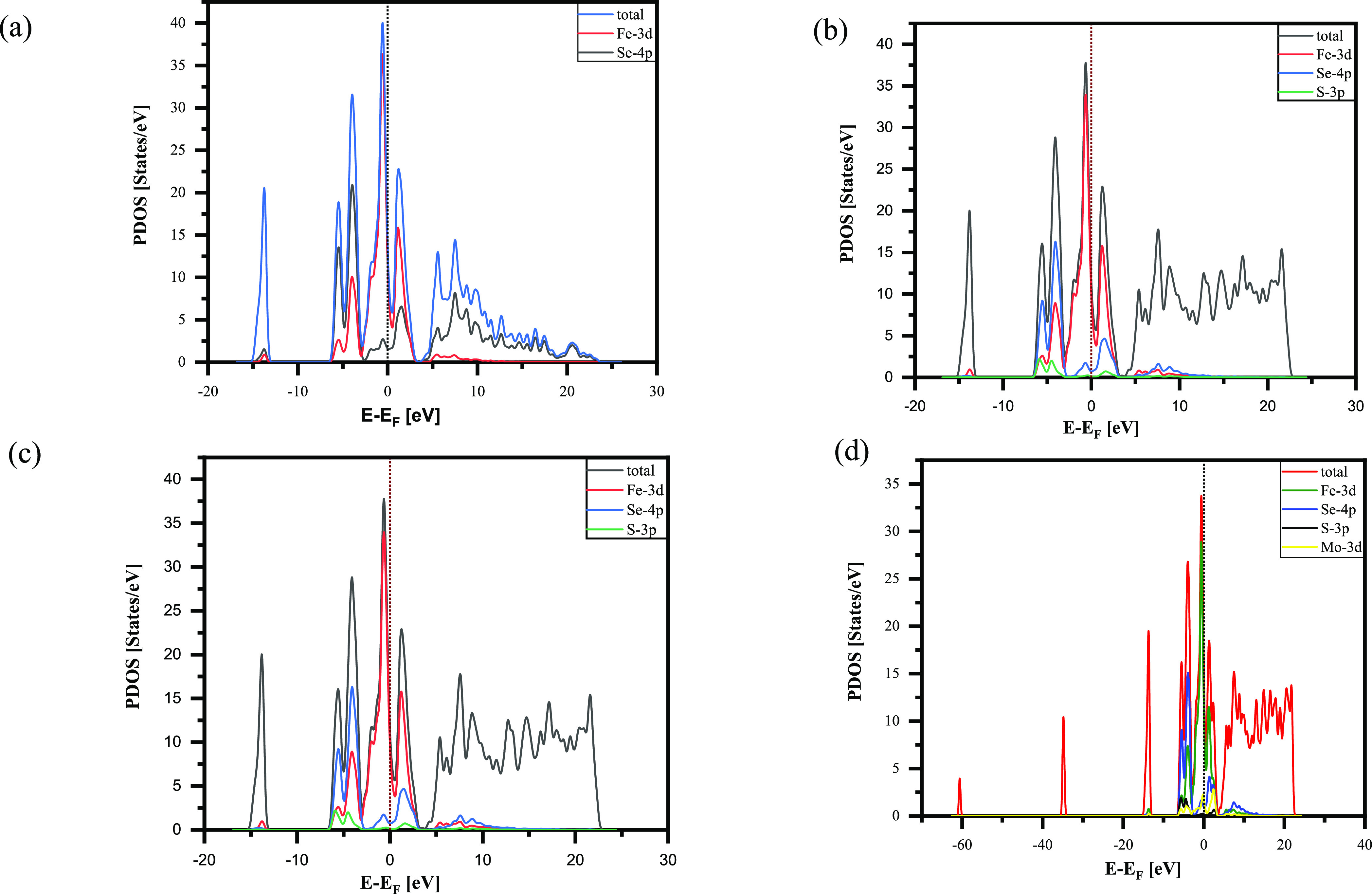
(a) Total density of state for the FeSe unit cell and partial density of state for the 3d orbital of Fe and 4p orbital of Se. (b) Total density of state for FeSe_0.875_S._0125_ and partial density of state for the 3d orbital of Fe, 4p orbital of Se, and 3p orbital of sulfur. (c, d) Total density of state for Mo_0.125_Fe_0.875_Se_0.875_S_0.125_ and.

The partial DOS for pure FeSe, Mo, and sulfur doped is shown in Figure 7a,b. By comprising the pure FeSe with doping with 12.5% of S in panel b, we can observe the increase of total density of state at the Fermi level (*E*_F_), with a small shift toward the high energy. This observation aligns with previous expectations from the literature review.^15^ However, for Mo substitution, the total DOS at the Fermi level changes significantly, increasing from approximately 11.8 to 15.8 states/eV. This change is quite substantial. These results suggest that Mo is different compared to other transition metals and can negatively impact superconductivity at the same doping percentage. Conversely, this correlates with the enhancement of superconductivity in FeSe_0.875_S_0.125_, which is consistent with the phonon mechanism of superconductivity. Regarding the different positions of Mo in the crystal structure, it was observed that they do not significantly affect the density of states. However, there are changes in the partial density of states, particularly in the Fe-3d orbitals, which increase with Mo substitution. To gain a better understanding of the superconducting properties of Mo_0.125_Fe_0.875_Se_0.875_S_0.125_, it is crucial to analyze the changes in the electronic density of states near the Fermi level (*E*_F_) upon Mo insertion. Comparison of the calculated DOS for Mo_0.125_Fe_0.875_Se_0.875_S_0.125_ and the parent compound FeSe yields the main results: the insertion of Mo leads to a significant restructuring of the DOS. This goes beyond the changes expected on the basis of a rigid band picture and the increasing of the DOS at *E*_F_. The presence of a DOS spike at the Fermi level is predicted to correspond with the observed increased critical temperature of superconductivity.

Table 1 shows how the charge on Fe atoms increases by Mo doping on FeSe_0.875_S_0.125_ from −24 to −25, which indicated the efficiency of hole doping in the Fe site and increase in the number of electrons around Fe atoms. This is related to the chemical pressure induced by the dopants. The charge transfer increases due to the decrease of the distance between the atoms. This describes the decrease of bond length between Fe and Se from 2.47388 to 2.339.1 Å .

**Table 1:** Change in Bond Length and Charge on Se and Fe by Mo and S Dopants

samples	bond length Fe–Fe	bond length Fe–Se	Fe charge	Se charge
Mo_0.125_Fe_0.875_Se_0.875_S_0.125_	2.66767	2.33901	–0.2500	0.2900
FeSe_0.875_S_0.125_	2.67188	2.43233	–0.2400	0.2500
FeSe	2.66954	2.47388	–0.120	0.120

## 4 Conclusions

We have investigated the effect of the Mo substitution in the Fe site for hole doping in the FeSe system in both experimental and computational studies. According to experiments, it is known that the Mo atom could not enter the FeSe system due to the large atomic radius compared with the Fe atom, so all routes for substitution are not successful. Here, we provide a new way to study the effect of Mo doping by doping Mo with a Mo/Fe molar ratio of 0.1:0.9 in FeSe_0.9_S_0.1_. The composition to be investigated is Mo_0.1_Fe_0.9_Se_0.9_S_0.1_. The polycrystalline samples were prepared by a one-step solid-state reaction. Our results show that instead of Mo substitution, the MoS_2_ is formed layer-by-layer with FeSe. The computational calculations of DOS and band structure by the Materials Studio program in CASTEP code confirm the high effect of Mo substitution on electronic structure by increasing the DOS at the Fermi level, which indicted the enhancement of *T*_c_ in Mo_0.125_Fe_0.875_Se_0.875_S_0.125_. We have succeeded also in preparing FeSe in the tetragonal phase and determining how the structure is sensitive to doping or the molar ratio.
